# Esophageal microflora in esophageal diseases

**DOI:** 10.3389/fcimb.2023.1145791

**Published:** 2023-05-19

**Authors:** Qian Zou, Lijuan Feng, Xunchao Cai, Yun Qian, Long Xu

**Affiliations:** Department of Gastroenterology and Hepatology, Shenzhen University General Hospital, Shenzhen, China

**Keywords:** esophageal diseases, microbiota, gastroesophageal reflux, Barrett’s esophagus, eosinophilic esophagitis, esophageal cancer

## Abstract

With the development of endoscopic technology, an increasing number of patients with esophageal disease are being diagnosed, although the underlying pathogenesis of many esophageal diseases remains unclear. In recent years, a large number of studies have demonstrated that the occurrence and development of various intestinal diseases were related to intestinal flora. As a result, researchers have shifted their focus towards investigating esophageal flora to better understand the pathogenesis, early diagnosis, and treatment of esophageal diseases. This paper reviewed the normal esophageal flora and the changes of esophageal flora under different esophageal disease states. It was observed that there are distinct differences in the composition of esophageal microflora among Gastroesophageal Reflux, Barrett’s esophagus, eosinophilic esophagitis and normal esophagus. The normal esophageal flora was dominated by gram-positive bacteria, particularly *Streptococcus*, while the esophageal flora under esophagitis was dominated by gram-negative bacteria. Furthermore, the diversity of esophageal flora is significantly decreased in patients with esophageal cancer. Several potential microbial biomarkers for esophageal cancer have been identified, among which *Fusobacterium nucleatum* showed a close association with esophageal squamous cell carcinoma’s pathological stage and clinical stage.

## Introduction

In recent years, there has been increasing interest in studying human microorganisms, and it is now widely recognized that these microorganisms are closely linked to various human diseases (Team NIHHMPA, 2019). However, compared to other parts of the body, the esophageal microflora has received limited attention and keep understudied. Moreover, the correlation between esophageal microflora and esophageal diseases is still largely unclear. Fortunately, with the improvement of sampling technology and the rapid progress of high-throughput sequencing technology, researches on esophageal microflora have gradually increased. Through these studies, we now have a preliminary understanding of the common microflora in a healthy esophagus (Pei et al., 2004; Yang et al., 2009). Furthermore, studies have shown that esophageal diseases such as gastroesophageal reflux disease (GERD), Barrett’s esophagus (BE), Eosinophilic esophagitis (EoE) and esophageal carcinoma (EC) all have their own unique microflora profiles (Paull and Yardley, 1988; Tian et al., 2015; Nardone et al., 2017; Polyzos et al., 2018). Here, we will review the changes of esophageal microflora table under healthy esophagus and different esophageal disease states, aiming to enhance the understanding of the differences of esophageal microflora under different esophageal disease states. This will help to elucidate the correlation between esophageal microflora and esophageal diseases, and may provide new avenues to prevent or treat esophageal diseases.

## Esophageal microflora of the normal esophagus

The human microflora has been a hot topic of research in recent years, the studies on intestinal microflora emerge endlessly and a lot of remarkable results have been achieved. However, studies on the esophageal microflora have been limited due to the constraints in sampling techniques, isolation and culture methods. Even after a long time, there is no definitive answer on whether there are long-term colonized microorganisms in the esophagus. Many scientists even believe that the esophagus is an environment for asepsis or the transient passage of bacteria. It was not until the early 1980s that the esophagus was confirmed for the first time that it was not a completely sterile environment through the traditional isolation and culture method (Lau et al., 1981; Finlay et al., 1982; Mannell et al., 1983). In 1998, Gagliardi et al. (1998) used the esophageal aspirate to obtain and culture the esophageal microflora, which showed that the most common bacteria in the esophagus was *Streptococcus viridans*. Because *S. viridans* was also grown in the oropharynx, the researchers thought that there might be some correlations between the esophageal microflora and the oropharyngeal microflora. Further investigations by Zilberstein et al. (2007) tested the esophageal microflora of 5 healthy adults with esophageal aspirate and culture, and the main bacteria they detected were *Streptococcus*, *Staphylococcus*, *Corynebacterium*, *Lactobacillus*, *Peptococcus*, among which *Streptococcus* is the most prevalent, with a percentage of 40%. However, although the above studies have helped people understand that the esophagus is not sterile, studies based on the above methods have seriously underestimated the complexity of the esophageal microflora due to the close adhesion of some bacteria to the mucosa and the challenges in culturing certain bacteria. In 2004, Pei et al. (2004) determined the colonized microflora in the normal distal esophagus by mucosal biopsy and 16S rRNA gene sequencing for the first time. They revealed that the normal esophageal microflora mainly consisted of six phyla: Firmicutes (mainly *Streptococcus*, *veillonella*, *Megasphaera*, *Granulicatella*, *Gemella*, *Clostridium* and *Bulleidia*), Bacteroidetes (mainly *Prevotella* and *Bacteroides*), Proteobacteria (mainly *Haemophilus*), Fusobacteria, Actinobacteria (mainly *Rothia* and *Actinomyces*), and Saccharibacteria. Among these phyla, the dominant phylum is Firmicutes (specifically *Streptococcus mitis*). This method of obtaining tissue through mucosal biopsy and then measuring microflora through gene sequencing opened another door to study the esophageal microflora. After that, 16S rRNA gene sequencing technology has become increasingly popular with the continuous progress of molecular technology, allowing for cost-effective large sample detection and quantitative characterization of microflora in complex biological mixtures, or even the entire community and its components (Metzker, 2010; Cox et al., 2013). In 2009, Yang et al. (2009) conducted distal esophageal biopsy sampling and 16S rRNA gene sequencing on 34 subjects, and finally divided the esophageal microflora into two categories, among which type I microflora mainly consisted of gram-positive bacteria predominantly belonging to *Streptococcus*, and was commonly found in normal esophagus. This finding was later verified by many research results. In 2013, Liu et al. (2013) took mucosal biopsy samples from the distal esophagus of 6 healthy subjects, and analyzed 147 cloned 16S rRNA sequences, they found that there were four phyla in the distal of normal esophagus of Japanese individuals: Proteobacteria (49%), Firmicutes (40%), Bacteroidetes (8%) and Actinomycetes (3%). There were eleven genera (≥3%) detected, including *Streptococcus* (21%), *Klebsiella* (10%), *Gemella* (6%), *Eubacterium* (5%), *Helicobacter* (4%), *Escherichia* (4%), *Haemophilus* (4%), *Granulicatella* (4%) *Citrobacter* (4%) *Prevotella* (3%) and *Bulleidia* (3%), with *Streptococcus* being the most prevalent. In summary, although there are limited studies on the healthy esophageal microflora, and most of them have been used as controls in esophageal diseases research with variations in sampling methods, detection techniques and even sampling sites, it is generally accepted that there is resident microflora in normal esophagus, mainly gram-positive bacteria. Among them, Firmicutes (*Streptococcus*) is the most common. Proteobacteria, Bacteroidetes and Actinobacteria are also mentioned. They play an important role in maintaining the normal environment of the esophagus (**Table 1**).

**Table 1:** Main findings about esophageal microflora of the normal esophagus.

Year & Study	Population	Sample Type	Location of sampling	Method	Main findings
Gagliardi et al. (1998)	30 subjects with normal esophagus	esophageal aspirate	The medial third of the esophagus	Culture	*S. viridans* (62.5%), *Streptococcus pneumoniae* (12.5%), *Non-Entcrococcus D group* *Streptococcus* (12.5%), *Staphylococcus aureus* (12.5%)
Pei et al. (2004)	4 subjects with normal esophagus	mucosal biopsy	distal esophagus	16S rRNA	6 phyla: Firmicutes, Bacteroidetes, Proteobacteria, Fusobacteria, Actinobacteria, and Saccharibacteria,
Zilberstein et al. (2007)	5 subjects with normal esophagus	esophageal aspirate	proximal end of esophagus	Culture	*Streptococcus* (40%), *Staphylococcus* (20%), *Corynebacterium* (10%), *Lactobacillus* (10%), *Peptococcus* (10%)
Yang et al. (2009)	12 subjects with normal esophagus;12 subjects with esophagitis;10 subjects with BE	mucosal biopsy	distal esophagus	16S rRNA	divided the esophageal microflora into two categories, type I microflora mainly consisted of gram-positive bacteria (dominated by *Streptococcus*) mainly appeared in normal esophagus.
Liu et al. (2013)	6 subjects with normal esophagus;6 subjects with RE;6 subjects with BE	mucosal biopsy	distal esophagus	16S rRNA	4 phyla:Proteobacteria (49%), Firmicutes (40%), Bacteroidetes (8%) and Actinomycetes (3%). 11 genera: *Streptococcus* (21%), *Klebsiella* (10%), *Gemella* (6%), *Eubacterium* (5%), *Helicobacter* (4%), *Escherichia* (4%), *Haemophilus* (4%), *Granulicatella* (4%) *Citrobacter* (4%) *Prevotella* (3%) and *Bulleidia* (3%).

## Esophageal microflora of the gastroesophageal reflux and Barrett’s esophagus

Gastroesophageal reflux (GERD) is characterized by distressing symptoms such as acid reflux and heartburn, as well as complications including bleeding and esophageal stenosis caused by the reflux of gastric contents into the esophagus. Furthermore, chronic gastroesophageal reflux can cause the stratified squamous epithelium that covers in the distal esophagus to be replaced by columnar epithelium, leading to the development of Barrett’s esophagus (BE). Barrett’s esophagus is recognized as a precancerous disease associated with esophageal adenocarcinoma. Therefore, many scientists hope to find strategies to prevent the occurrence of esophageal adenocarcinoma by studying the pathogenesis of GERD and Barrett’s esophagus. In the past, it was believed that GERD and Barrett’s esophagus were triggered by the inflammation of the esophageal mucosa due to exposure to gastric acid or bile. Consequently, the clinical treatment of GERD and Barrett’s esophagus mainly focused on inhibiting gastric acid, protecting esophageal mucosa and promoting peristalsis of esophagus and stomach. However, in 2009, a study linked esophagitis to changes in the esophageal microflora. Yang et al. (2009) divided the esophageal microflora into two types, and they believed that the type II microflora, composed mainly of gram-negative or anaerobic bacteria such as *Veillonella*, *Prevotella*, *Haemophilus*, *Campylobacter, Porphyromonas, Neisseria*, *Granulicatella*, *Fusobacterium*, *Rothia* and *Actinomyces*, was closely associated with esophageal diseases such as GERD and Barrett’s esophagus, However, based on current data, it is impossible to determine whether the type II microflora plays a pathogenic role in GERD or Barrett’s esophagus, or whether acid reflux changes the esophageal microflora by affecting acid-sensitive bacteria in the esophagus. In 2013, Liu et al. (2013) studied the esophageal microflora of 6 patients with reflux esophagitis through 16S rRNA gene sequencing. They found that Proteobacteria (43%) was the most prevalent phylum in reflux esophagitis, followed by Firmicutes (33%), Fusobacteria (10%), Bacteroidetes (10%), Saccharibacteria (2%) and Actinomyces (2%). Eleven genera (≥3%) were detected in patients with reflux esophagitis, mainly including *Streptococcus* (20%), *Pasteurella* (10%), *Haemophilus* (9%), *Fusobacterium* (9%), *Klebsiella* (9%), *Prevotella* (5%), *Neisseria* (4%), *Veillonella* (3%), *Bacillus* (3%) and *Helicobacter* (3%). The researchers also investigated the esophageal microflora of 6 patients with Barrett’s esophagus. The most prevalent phylum in Barrett’s esophagus was Firmicutes (55%), followed by Proteobacteria (20%), Bacteroidetes (14%), Fusobacteria (9%) and Actinobacteria (2%). Similarly, 11 genera (≥3%) were detected in patients with Barrett’s esophagus, mainly including *Veillonella* (19%), *Prevotella* (12%), *Streptococcus* (11%), *Fusobacterium* (9%), *Lactobacillus* (4%), *Actinobacillus* (4%), *Neisseria* (4%), *Helicobacter* (4%), *Gemella* (4%), *Achromobacter* (3%) and *Dialister* (3%). Notably, patients with Barrett’s esophagus had a lower proportion of *Streptococcus* compared to patients with reflux esophagitis and normal esophagus. In addition, *Fusobacterium*, *Neisseria* and *Veilonella* were commonly detected in patients with reflux esophagitis and Barrett’s esophagus, but not in subjects with normal esophagus, indicating differences in microflora among these esophageal conditions. Furthermore, Harris et al. (2015) studied the esophageal microflora of 8 subjects with GERD (4 treated and 4 untreated), and found that the average amount of bacteria in GERD patients was significantly increased compared to normal esophageal subjects. This increase was mainly represented by an increase in Firmicutes and a decrease in Proteobacteria. Similarly, Park et al. (2020) analyzed the esophageal microflora of 18 Non-erosive Gastrooesophageal Reflux Disease (NERD) patients through 16S rRNA gene sequencing, and found that the most common bacterial groups at the phylum level were Firmicutes, Proteobacteria and Bacteroidetes. At the level of genus, *Streptococcus*, *Haemophilus*, *Prevotella*, *Veillonella*, *Neisseria* and *Granulicatella* were more prevalent. This is consistent with the fingdings of Yang et al. (2009). In addition, Okereke et al. (2021) compared the esophageal microflora of 24 patients with Barrett’s esophagus and 40 GERD patients without Barrett’s esophagus, and found that there was a dramatic difference between the Barrett’s esophagus group and the GERD group in a variety of microorganisms (*Actinomyces*, *Prevotellapallens*, *Dialister*, *Streptococcus salivarius*, *Prevotella unspecified*, *Streptococcus unspecified*). With the increase of the length of Barrett’s column, the possibility of detecting various bacteria in the distal esophagus decreased, especially the possibility of detecting *Streptococcus*, *Neisseria*, *Leptotrichia*, *Dialister*, *Gemella*, *Veillonella*, *Corynebacterium*, *Rothia*, *Haemophilus* and *Prevotella*. The study of Zhou et al. (2020) showed that compared to the normal esophagus group, which had a higher level of gram-positive Firmicutes and Actinomyces, the composition of the NERD microflora has shifted from Fusobacteria and Actinobacteria to Proteobacteria and Bacteroidetes. The composition of microflora in reflux esophagitis and Barrett’s esophagus shifted from Firmicutes to gram-negative Fusobacteria and Proteobacteria, as revealed by 16S rRNA gene sequencing. However, it should be noted that bacteria detected through this method may be not viable and could have amplification deviations. As a result, some researchers still employ traditional culture techniques to measure esophageal microflora. Norder et al (Norder Grusell et al., 2018). measured esophageal microflora in 17 GERD subjects by brush sample, mucosal biopsy and AGAR culture. Alpha-*Streptococcus* was cultured in 71% (brush) and 76% (biopsy) of the distal esophagus, along with other common genera such as *Lactobacillus*, *Prevotella*, *Haemophilus*, *Clostridium* and *Neisseria*. However, not all studies demonstrate significant differences in the microflora of patients with esophagitis compared to those with a normal esophagus. Yu et al. (2019) compared the esophageal microflora of 17 healthy subjects and 32 patients with reflux esophagitis, and found no significant differences between two groups. At the phylum level, only Bacteroidetes differed between groups, with relatively low abundance in the reflux esophagitis group. No significant differences were observed at the family and genus levels. Although the findings of microflora measurement in patients with GERD and Barrett’s esophagus between studies are varied, most studies demonstrated that there are differences in esophageal microflora among GERD, Barrett’s esophagus and normal esophagus. Specifically, gram-negative bacteria are more abundant in GERD and Barrett’s esophagus, and the diversity of microflora is lower in Barrett’s esophagus. The proportion of *Streptococcus* is reduced in Barrett’s esophagus compared to that in GERD and normal esophagus. Furthermore, *Fusobacterium*, *Neisseria* and *Veilonella* were commonly detected in patients with reflux esophagitis and Barrett’s esophagus, but not in subjects with normal esophagus. These findings provide insights for further understanding of the pathological mechanisms of GERD and Barrett’s esophagus and for predicting the transformation of Barrett’s esophagus into esophageal adenocarcinoma through changes in esophageal microflora. Proteobacteria, Bacteroidetes and Actinobacteria are also mentioned as important phyla that play a role in maintaining the normal esophageal environment. (**Table 2**)

**Table 2:** Main findings about esophageal microflora of Gastroesophageal reflux (GERD) and Barrett’s esophagus (BE).

Year&Study	Population	Sample Type	Location of sampling	Method	Main findings
Yang et al. (2009)	12 subjects with normal esophagus;12 subjects with esophagitis; 10 subjects with BE	mucosal biopsy	distal esophagus	16S rRNA	divided the esophageal microflora into two categories, type II microflora, composed mainly of gram-negative bacteria or anaerobic bacteria, was closely associated with esophageal diseases such as GERD and Barrett’s esophagus
Liu et al. (2013)	6 subjects with normal esophagus; 6 subjects with RE;6 subjects with BE	mucosal biopsy	distal esophagus	16S rRNA	RE: Proteobacteria (43%), Firmicutes (33%), Fusobacteria (10%), Bacteroidetes (10%), Saccharibacteria (2%) and actinomyces (2%) BE:5 phyla: Firmicutes (55%), Proteobacteria (20%), Bacteroidetes (14%), Fusobacteria (9%) and Actinobacteria (2%))
Harris et al. (2015)	8 subjects with GERD (4 untreated and 4 treated);37 subjects with EoE(11 untreated and 26 treated); 25 subjects with normal esophagus	Esophageal String Test (EST)	NA^a^	16S rRNA	GERD patient’s bacterial amount was significantly increased compared with normal esophageal subjects, mainly increased in Firmicutes and decreased in Proteobacteria.
Norder Grusell et al. (2018)	17 subjects with GERD;10 subjects with EoE	cytology brush sample and mucosal biopsy	proximal esophagus and distal esophagus	Culture	α-*Streptococcus* was cultured in 71% (brush) and 76% (biopsy) of the distal esophagus. *Lactobacillus*, *Prevotella*, *Haemophilus*, *Clostridium* and *Neisseria* were also common
Yu et al. (2019)	17 subjects with normal esophagus; 32 subjects with RE	mucosal biopsy	distal esophagus	16S rRNA	No significant difference compared with normal esophagus
Park et al. (2020)	18 subjects with NERD	mucosal biopsy	NA^a^	16S rRNA	the most common bacterial groups at the phylum level were Firmicutes, Proteobacteria and Bacteroidetes. At the level of genus, *Streptococcus*, *Haemophilus*, *Prevotella*, *Veillonella*, *Neisseria* and *Granulicatella* were more common.
Zhou et al. (2020)	16 subjects with normal esophagus; 11 subjects with GERD; 20 subjects with RE; 17 subjects with BE;6. subjects with EAC	cytology brush sample and mucosal biopsy	proximal esophagus and distal esophagus	16S rRNA	NERD: shift from Fusobacteria and Actinobacteria to Proteobacteria and Bacteroidetes RE and BE: shift from Firmicutes to gram-negative Fusobacteria and Proteobacteria.
Okereke et al. (2021)	34subjects with BE;40 subjects with GERD	mucosal biopsy	proximal esophagus and distal esophagus	16S rRNA	With the increase of the length of Barrett’s column, the variety of bacteria in the distal esophagus decreased, especially *Streptococcus, Neisseria, Leptotrichia, Dialister, Gemella, Veillonella, Corynebacterium, Rothia, Haemophilus* and *Prevotella*

^a^ NA represents not available.

## Esophageal microflora of the eosinophilic esophagitis

Eosinophilic esophagitis is a chronic esophagitis characterized by eosinophil infiltration into the esophageal wall. Swallowing obstruction, food incarceration, and reflux symptoms are the most common clinical symptoms. Since eosinophilic esophagitis is an immune-related disease and its pathogenesis is not fully understood, it has been hypothesized that esophageal ecological disorders are the trigger for EoE pathology (Jensen and Dellon, 2018). Additionally, environmental factors related to living conditions may also increase EoE susceptibility by causing changes in the esophageal microflora (Jensen et al., 2018). Therefore, researchers have attempted to explore the diagnosis and treatment of eosinophilic esophagitis by studying the esophageal microflora. In 2015, Harris et al. (2015) measured the esophageal flora of 37 EoE subjects (11 untreated and 26 treated), 8 GERD subjects and 25 normal subjects. The results showed no significant difference in α diversity between EoE subjects and the control group, either in normal subjects. In patients with active or treated EoE, Bacteroidetes, Firmicutes, Fusobacterium, and Proteobacteria were dominant, but the average bacterial load detected in all EoE subjects was significantly higher than in normal subjects. There was a significant increase in *Haemophilus* in untreated EoE compared to normal control subjects, and the esophageal microflora of untreated EoE subjects shifted from predominantly gram-positive bacteria (mainly Firmicutes) to gram-negative bacteria (mainly Proteobacteria). This is consistent with Yang et al.’s view that TypeI bacteria were more common in the normal esophagus and Type II bacteria were more common in patients with esophagitis (Yang et al., 2009). In another study by Benitez et al. (2015), the esophageal microflora of 33 children with EoE and 35 non-EOE children was analyzed, and the results showed that compared to non-EOE children, the abundance of Proteobacteria (mainly *Corynebacterium* and *Neisseria*) in EoE patients was significantly increased, while Firmicutes were more prevalent in non-EOE children. Additionally, they found that the intake of highly allergenic foods could significantly increase the abundance of *Ganulicatella* and *Campylobacter* in the esophagus of children with eosinophilic esophagitis. In 2018, a study on GERD and EoE by Norder et al (Norder Grusell et al., 2018). showed that bacteria were present in the distal esophagus of all EoE subjects. Compared to GERD subjects and healthy subjects, patients with EoE exhibited a more diverse esophageal microflora. Through the results, they found that *S. viridans* was the most common bacteria in EoE patients. The study found an increase in *Haemophilus* in patients with EoE compared to patients with GERD, and *Prevotella* was more prevalent in subjects with EoE. Laserna-Mendieta et al. (2021) studies on active EoE, which showed that inflammation in EoE did not significantly alter the α-diversity of esophageal microflora. The dominant phylum in both EoE and non-EoE subjects was Firmicutes (65%), followed by Proteobacteria (18%) and Bacteroidetes (9%). *Streptococcus* was the dominant genus. Compared to the control group, the abundance of Proteobacteria in EoE patients was slightly decreased, while the abundance of Bacteroidetes was increased. However, not all the research shows that there were significant differences between EoE subjects and controls. In a study of 24 adult EoE and 25 adult non-EOE subjects, Johnson et al. (2021) reported no significant differences in esophageal microflora between newly diagnosed adult EoE cases and non-EOE controls, or between EoE cases. As eosinophilic esophagitis is a relatively rare esophageal disease, there are limited studies on the esophageal microflora of eosinophilic esophagitis. Besides, because the prone population of eosinophilic esophagitis includes both adults and children. There still has no definitive conclusion on the esophageal microflora of eosinophilic esophagitis. However, combined with the existing research results, the microflora of EoE patients demonstrated a shift from gram-positive bacteria to gram-negative bacteria, particularly Proteobacteria. Several studies have reported that Proteobacteria increased significantly in the esophagus of EoE patients, which is more pronounced in untreated EoE patients (**Table 3**).

**Table 3:** Main findings about Esophageal microflora of eosinophilic esophagitis.

Year&Study	Population	Sample Type	Location of sampling	Method	Main findings
Harris et al. (2015)	37 subjects with EoE;11 untreated and 26 treated); 8 subjects with GERD(4 untreated and 4 treated); 25 subjects with normal esophagus	Esophageal String Test (EST)	NA^a^	16S rRNA	the esophageal microbiota of untreated EoE subjects changed from predominantly gram-positive bacteria (mainly Firmicutes) to gram-negative bacteria (mainly Proteobacteria).
Benitez et al. (2015)	33 children with EoE;35 children without EOE	mucosal biopsy	NA^a^	16S rRNA	the abundance of Proteobacteria (mainly *Corynebacterium* and *Neisseria*) in EoE patients was significantly increased, intake highly allergenic foods could increase the abundance of *Ganulicatella* and *Campylobacter* of children with EoE
Norder Grusell et al. (2018)	10 subjects with EoE;17 subjects with GERD	cytology brush sample and mucosal biopsy	proximal esophagus and distal esophagus	Culture	*S. viridans* was the most common bacteria in EoE; *Haemophilus* and *Prevotella* were more common in subjects with EoE
Laserna-Mendieta et al. (2021)	30 subjects with active EoE; 10 subjects with normal esophagus	mucosal biopsy	both upper and lower esophageal thirds	16S rRNA	Firmicutes (65%); Proteobacteria (18%) and Bacteroidetes (9%). *Streptococcus* is the dominant genus. the abundance of Proteobacteria decreased, while the abundance of Bacteroidetes increased
Johnson et al. (2021)	24 subjects with EoE;25 subjects with normal esophagus	mucosal biopsy	mid-esophageal	16S rRNA	no significant differences in the esophageal microflora between newly diagnosed EoE cases and non-EOE controls, or between EoE cases

^a^ NA represents not available.

## Esophageal microflora of the esophageal cancer

With the rising incidence and mortality rates of esophageal cancer, clinicians and researchers have paid increasing attention to this disease in recent years. However, the early diagnosis and treatment of this disease have remained challenging, with many esophageal cancers cannot be found until they present with symptoms such as dysphagia or odynophagia (Cook et al., 2009; Abnet et al., 2018). The etiology and pathogenesis of esophageal cancer are still not fully understood, making it imperative to uncover potential biomarkers that can be clinically applied. With the deepening of research on human microbes, lots of evidences show that human microbes play a vital role in the occurrence and development of various malignant tumors (Rajagopala et al., 2017; Helmink et al., 2019; Matson et al., 2021; Sepich-Poore et al., 2021). Jiang et al.’s study (Jiang et al., 2021) on Esophageal Squamous Cell Carcinoma (ESCC) found there were significant differences in microbial diversity between ESCC subjects and healthy controls. The species and abundance of the esophageal microflora in patients with ESCC decreased significantly, which means, their bacterial diversity decreased. Specifically, at the phylum level, ESCC patients showed a decrease in Fusobacteria compared to the control group, and at the genus level, they detected a decrease in *Faecalibacterium*, *Curvibacter*, *Bacteroides* and *Blautia*. In comparison to the esophagitis group, the ESCC group demonstrated fewer *Faecalibacterium*, *Blautia*, *Bacteroides*, but increased *Streptococcus*. From normal control group to esophagitis group to ESCC group, *Megamonas*, *Collinsella*, *Roseburia*, and *Ruminococcus_2* showed a gradual decline. Using multi-level LEfSe analysis, potential microbe biomarkers for ESCC, such as *Actinobacillus*, *Peptostreptococcus*, *Streptococcus*, *Prevotella* and *Fusobacterium* were identified. Another study by Lin et al. (2022) found that the relative abundances of Proteobacteria, Fusobacteria, Bacteroidetes and Firmicutes were similar between tumors and adjacent tissues by sequencing the genes of tumor tissues and adjacent tissues of 120 ESCC patients. Within the class α Proteobacteria, Rhizobium and Sphingomycetes were enriched in tumor tissues, while Rhodospirillles was enriched in tumor adjacent tissues. Additionally, Saccharibacteria and its bacterial branches, including *H. pylori*, were also higher in adjacent tissues of the tumor. The study also revealed decreased esophageal bacterial diversity in ESCC patients. A study by Zhang et al. (2022) on the microflora of ESCC tumors and non-tumor tissues in the esophagus showed that *Brevibacillus* and *Treponema* were more commonly present in tumor tissues at N1 and N2 stages, respectively, while *Acinetobacter* was more common in tumor tissues at T3 stages. *Fusicatenibacter* was more common in T2 stage non-tumor tissues, while *Corynebacterium*, *Cupriavidus*, *Saccharimonadaceae-TM7x* and *Aggregatibacter* were more commonly found in T4 stage non-tumor tissues. In addition, more bacteria were associated with base excision repair in tumor tissues, whereas more with nitrotoluene degradation in non-tumor tissues. Different tumor stages can be associated with distinct major bacterial taxa, suggesting the potential involvement of these bacteria in the occurrence and development of ESCC. These findings may contribute to the identification of bacterial markers to predict, diagnose and even treat ESCC. Peter et al. (2020) compared paired samples (disease and non-disease) composed of normal esophagus (n=10), intestinal metaplasia (IM n=10), low-grade dysplasia (LGD n=10), high-grade dysplasia (HGD n=10) and adenocarcinoma (EAC n=10) to analyze the differences in esophageal microflora under different disease states. Results showed that compared to healthy controls, *Planctomycetes* and *Crenarchaeota* were significantly decreased in disease tissues, particularly in the HGD group and the adenocarcinoma group, and *Balneola* was also reduced in different disease groups, particularly in HGD group. *Planctomyces*, *Nitrosopumilus* and *Siphonobacter* in the diseases were also significantly decreased compared to the healthy control group. Li et al. (2021a) divided 276 subjects into 4 groups according to pathology: normal group (n=82), low-grade dysplasia group (LGD n=60), high-grade dysplasia group (HGD n=64) and esophageal squamous cell carcinoma group (ESCC n=70). They collected saliva and samples of the esophageal chest segment with a sterile brush. The study revealed that bacterial diversity in saliva and sterile brush samples decreased with the progression of disease. *Granulicatella*, *Leptotrichia*, *Streptococcus*, *Rothia*, *Schaalia* and *Gemella* were identified as main biomarkers in low grade dysplasia, while *Lactobacillus* was identified as the main biomarker in high grade dysplasia. *Bosea*, *Gemella*, *Lactobacillus* and *Solobacterium* were identified as the main biomarkers in ESCC. Zhou (Zhou et al., 2020) observed a unique esophageal adenocarcinoma (EAC) microflora composed of a high abundance of lactic acid-producing bacteria (mainly *Lactobacillus*, *Bifidobacterium*, *Staphylococcus* and *Streptococcus*), and they believed that this microflora may promote carcinogenic effects through maladjusted lactic acid metabolism. Hu et al. (2020) found that compared to normal esophageal tissues, the diversity and distribution of bacteria in esophageal cancer tissues were reduced and disordered. *Proteus*, *Fusobacterium*, and *Bacteroides* were the dominant bacteria at the genus level in ESCC mucosal tissues, while *Prevotella*, *Klebsiella*, *Clostridia*, *Delftia*, *Streptococcus* and *Serratia* had high abundance, belonging to the dominant genus in esophageal cancer tissue at the subordinate level. Although no significant correlation was found between different locations and stages of ESCC with esophageal microflora, differences in the type of ESCC and the presence or absence of lymph node metastasis could affect the structure of esophageal microflora. After lymph node metastasis, the number of Proteobacteria in esophagus decreased significantly, while the number of Bacteroidetes increased significantly. Shen et al. (2022) sequenced the tumor tissues and adjacent non-tumor tissues from 19 patients with ESCC, revealing that Proteobacteria, Firmicutes, Actinobacteria, Deinococcus-Thermus, and Bacteroidetes were the most common bacteria found in tumor tissues and adjacent non-tumor tissues. *Streptococcus* (6.93%) was the most abundant genus in tumor tissues, while *Labrys* (11.1%) was the most abundant genus in adjacent non-tumor tissues. Notably, the complexity of microbial interactions in adjacent non-tumor tissues is stronger than that in tumor tissues, suggesting that microbial interactions may play an important role in maintaining the normal microenvironment of tissues. Kovaleva et al. (2021) reported a significant correlation between bacterial load and tumor interstitial phenotype, with gram-positive bacteria dominating over gram-negative bacteria in the group with high CD206+ macrophage content. Furthermore, they suggested that ESCC could be divided into two groups, both of the which had a high content of CD206+ macrophages. However, the first group was dominated by gram-positive bacteria and showed a poor prognosis, whereas the second group was characterized by a low gram-positive bacterial load and showed a better prognosis. These findings highlight the association between gram-positive bacterial load and ESCC prognosis, and suggest that combining tumor microflora with other matrix markers may have prognostic significance for ESCC. Li et al. (2020) divided the subjects into five groups: normal group (n=70), esophagitis group (n=70), low-grade intraepithelial neoplasia group (LGIN n=70), high-grade intraepithelial neoplasia group (HGIN n=19) and ESCC group (n=7), and conducted gene sequencing of esophageal specimens from different groups. They found that *Neisseria*, *Haemophilus*, *Streptococcus*, and *Porphyromonas* were significantly different among the groups, with the abundance of *Streptococcus* decreased from normal to ESCC, while that of other genera increased. Finally, through various combination tests, it was found that the combination of *Streptococcus* and *Neisseria* could be used as a microbial prediction model for ESCC and its precancerous lesions. Yang et al. (2021) also showed that the microbial composition in tumor tissues of ESCC patients was significantly different from that in normal esophageal tissues. Specifically, in ESCC patients, the observed decreased microbial diversity and decreased abundance of Bacteroidetes, Spirochaetes and Fusobacterium. Taking these microbial groups as the imbalance index, it can be found that the imbalance flora can well distinguish ESCC from normal esophagus. In addition, Li et al. (2021b) and Yamamura et al. (2016) focused specifically on *Fusobacterium nucleatum* in ESCC tumor tissues, and found that the relative abundance of *F. nucleatum* in tumor tissues was significantly higher than that in paired normal tissues, and a higher positive rate of *F. nucleatum* could be observed in tumors with metastasis compared to tumors without metastasis. Moreover, the cancer specific survival was shortened and the cancer specific mortality was significantly increased in patients with *F. nucleatum* positive, indicating a close relationship between F. nucleatum, ESCC pT stage, and clinical stage. The researchers suggested that the abundance of *F. nucleatum* may be used in combination with other indicators to predict ESCC metastasis and prognosis (**Table 4**).

**Table 4:** Main findings about Esophageal microflora of esophageal cancer.

Year&Study	Population	Sample Type	Location of sampling	Method	Main findings
Yamamura et al. (2016)	325 subjects with esophageal cancer(EC)	Formalin-fixed, paraffin-embedded EC specimens	NA^a^	qPCR	*F. nucleatum–positive patients had shorter cancer-specific survival and* higher cancer-specific mortality; *F. nucleatum*–positive patients with SCC had lower cancer-specific survival;
Peter et al. (2020)	10 subjects with normal esophagus;10 subjects with IM;10 subjects with LGD;10 subjects with HGD;10 subjects with EAC	mucosal biopsy	Barrett’s/EAC and visibly unaffected esophagus	16S rRNA	The *Planctomycetes* and the *Crenarchaeota* were reduced in disease tissues, particularly in HGD and EAC group; *Balneola* was reduced in different disease groups, particularly in HGD group; *Planctomyces*, *Nitrosopumilus* and *Siphonobacter* were reduced in different disease groups,
Zhou et al. (2020)	16 subjects with normal esophagus;11 subjects with GERD;20 subjects with RE; 17 subjects with BE;6. subjects with EAC	cytology brush sample and mucosal biopsy	proximal esophagus and distal esophagus	16S rRNA	EAC: A unique microflora composed of a large number of lactic acid-producing bacteria (mainly *Lactobacillus*, *Bifidobacterium*, *Staphylococcus* and *Streptococcus*)
Hu et al. (2020)	54 subjects with ESCC	surgical resection	NA^a^	NA^a^	*Proteus*, *Fusobacterium*, and *Bacteroides* were the dominant bacteria in ESCC mucosal tissues at the genus level; after lymph node metastasis, the number of Proteobacteria decreased, while the number of Bacteroidetes increased
Li et al. (2020)	70 subjects with normal esophagus; 70 subjects with ES;70 subjects with LGIN;19 subjects with HGIN; 7 subjects with ESCC	cytology brush sample and mucosal biopsy	middle esophagus (normal esophagus)/only the lesion	16S rRNA	*Neisseria*, *Haemophilus*, *Streptococcus*, and *Porphyromonas* were significantly different among each group; the abundance of *Streptococcus* decreased from normal to ESCC.
Jiang et al. (2021)	32 subjects with ESCC;15 subjects with ES; 21 subjects with normal esophagus;	surgical resection	NA^a^	16S rRNA	ESCC patients detected a decrease in Fusobacteria at the phylum level, and a decrease in *Faecalibacterium*, *Curvibacter*, Bacteroides and *Blautia* at the genus level; *Megamonas*, *Collinsella*, *Roseburia*, and *Ruminococcus_2* showed a gradual decline from normal control group to esophagitis group to ESCC group
Li et al. (2021a)	82 subjects with normal esophagus; 60 subjects with LGD;64 subjects with HGD;70 subjects with ESCC	cytology brush sample	a location 25cm from the central incisor (normal esophagus)/only the lesion	16S rRNA	*Granulicatella*, *Leptotrichia*, *Streptococcus*, *Rothia*, *Schaalia* and *Gemella* are the main biomarkers in low grade dysplasia;*Lactobacillus* is the main biomarker in high grade dysplasia; *Bosea*, *Gemella*, *Lactobacillus* and *Solobacterium* were the main biomarkers in ESCC
Kovaleva et al. (2021)	48 subjects with ESCC	surgical resection	NA^a^	16S rRNA	ESCC could be divided into two groups. the first group was dominated by gram-positive bacteria and showed a poor prognosis. The second group was characterized by a low gram-positive bacterial load and showed a better prognosis
Li et al. (2021b)	41 subjects with ESCC	esophageal tissue wax blocks	NA^a^	NA^a^	The top 6 phyla were *Actinobacteria*, *Bacteroidetes*, *Firmicutes*, *Bacteroidetes*, *Fusobacteria*, *Proteobacteria* and *Spirochaetes*; the abundance of *F. nucleatum* was highly correlated with the pT and clinical stages
Yang et al. (2021)	18 subjects with ESCC;11 subjects with normal esophagus	mucosal biopsy or surgical resection	NA^a^	16S rRNA	ESCC: the microbial diversity of the esophageal microflora decreased, and the abundance of Bacteroidetes, Spirochaetes and Fusobacterium decreased
Lin et al. (2022)	120 subjects with primarily ESCC	surgical resection	NA^a^	16S rRNA	In class α Proteobacteria, the Rhizobium and Sphingomycetes were enriched in tumor tissues, while the abundance of Rhodospirillles is higher in tumor adjacent tissues.
Zhang et al. (2022)	31 men with ESCC	surgical resection	NA^a^	16S rRNA	*Brevibacillus* and *Treponema* were more common in tumor tissues at N1 and N2 stages, respectively; *Acinetobacter* was more common in tumor tissues at T3 stages.; *Fusicatenibacter* was more common in T2 stage non-tumor tissues; *Corynebacterium*, *Cupriavidus*, *Saccharimonadaceae-TM7x* and *Aggregatibacter* were more common in T4 stage non-tumor tissues.
Shen et al. (2022)	19 subjects with ESCC	surgical resection	NA^a^	16S rRNA	Proteobacteria, Firmicutes, Actinobacteria, Deinococcus-Thermus and Bacteroidetes were the most common bacteria; at the genus level, the bacteria with the highest proportions in tumors was *Streptococcus* and the bacteria with the highest proportions in adjacent non-tumor tissues was *Labrys*.

^a^ NA represents not available.

## Conclusions

**Figure 1:**
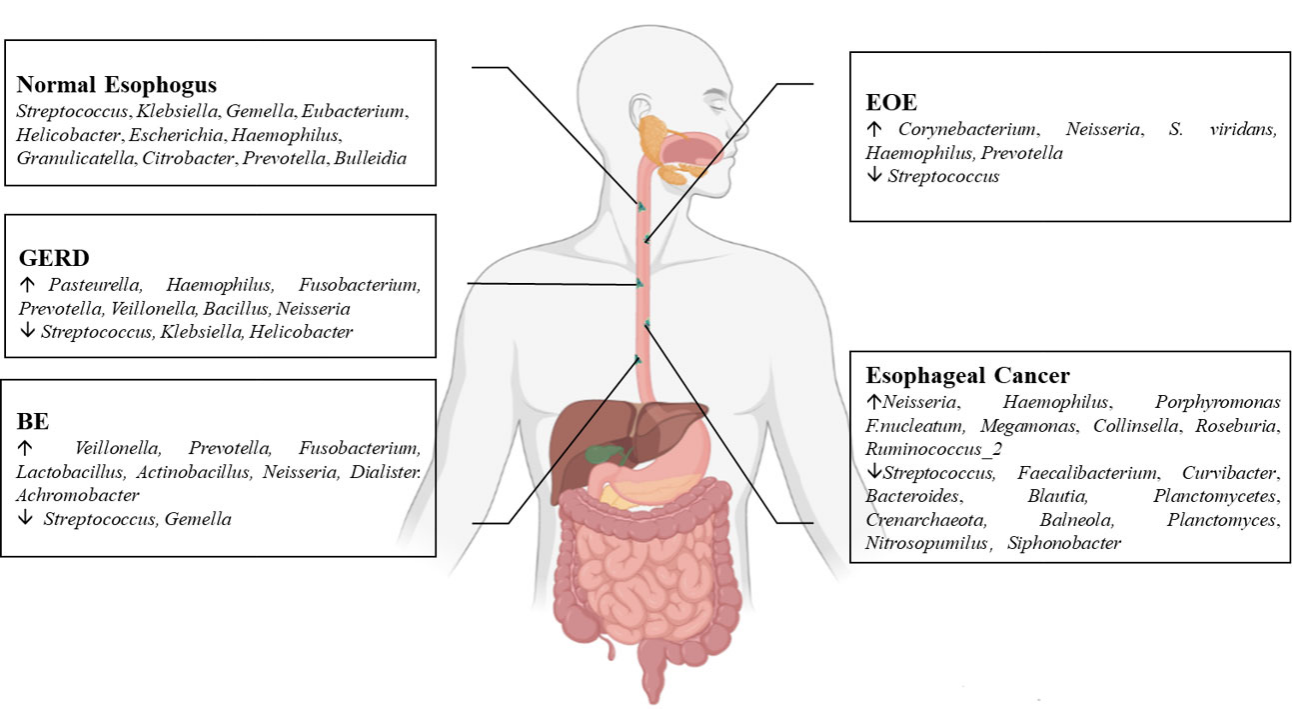
Changes of the esophageal microflora in various esophageal diseases.

**Figure 2:**
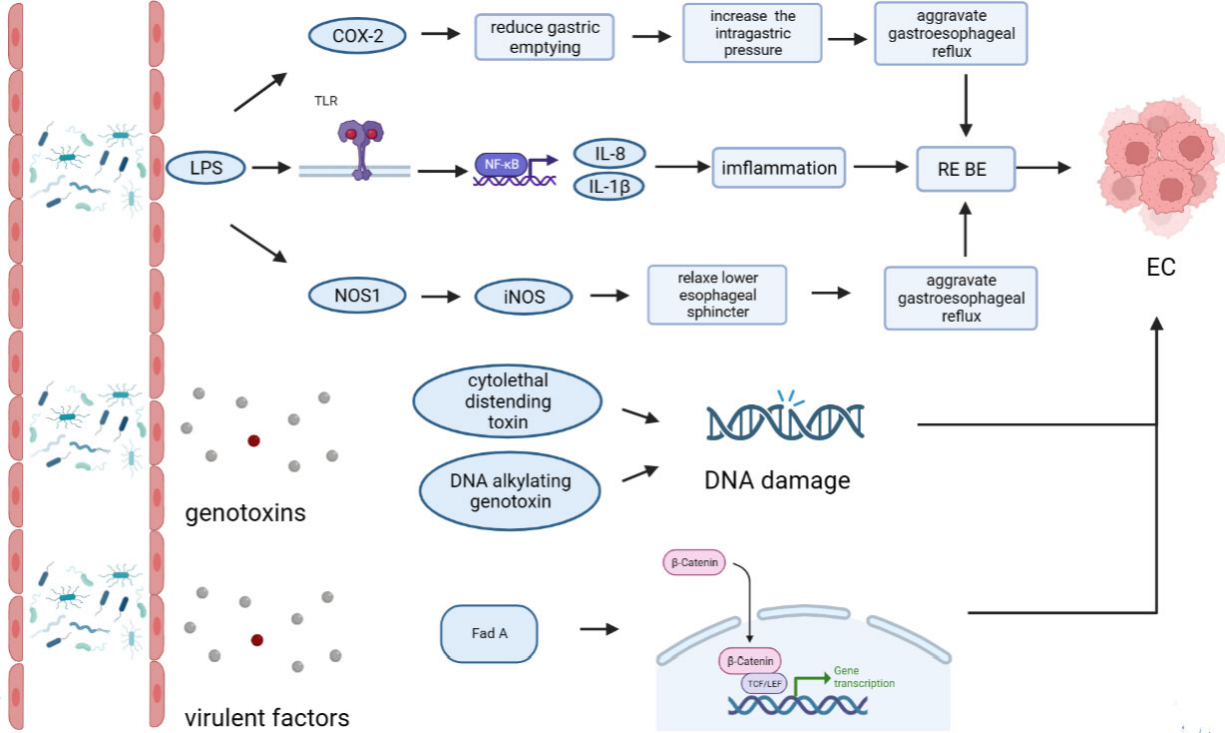
Mechanism of the esophageal microflora in esophageal diseases.

The esophageal microflora is gradually attracting attention in recent years, with a growing number of studies conducted on this topic. Current studies indicate that there is resident microflora in both the normal esophagus and the esophagus suffering from esophageal diseases, and the composition of the esophageal microflora varies among different diseases (**Figure 1**). As we known, the esophageal microflora is not completely consistent with that of any other part of human body, means that the esophageal microflora has its own uniqueness. But it has been observed that the taxon and composition of bacteria in the esophagus were similar to those in the oral cavity, suggesting that some esophageal bacteria come from the oral cavity, which may be related to the fact that bacteria in the oral cavity can migrate to the esophagus through swallowing and saliva (Mannell et al., 1983; Dewhirst et al., 2010; Yang et al., 2021). Additionally, the esophageal microflora is also influenced by refluxed gastric bacteria. In the normal esophagus, gram-positive bacteria dominate, including six phyla (Firmicutes, Bacteroidetes, Proteobacteria, Fusobacteria, Actinobacteria, Saccharibacteria). Among them, Firmicutes is the most dominant phylum, and *Streptococcus* was the most prevalent genus. However, in patients with esophagitis, including reflux esophagitis, Barrett’s esophagitis and eosinophilic esophagitis, gram-negative bacteria dominate, and the abundance of *Streptococcus* decreases. Lipopolysaccharide (LPS) present in gram-negative bacteria can activate Toll-like receptors (TLR), which further activate the NF-κB pathway, promoting the secretion of pro-inflammatory cytokine IL-8, IL-1β. This activation is a key step in the transformation of normal esophagus to BE or esophageal cancer (Pikarsky et al., 2004; Tsukamoto et al., 2018). LPS can also activate inducible nitric oxide synthase one (NOS1) and up-regulate iNOS, leading to the relaxation of the lower esophageal sphincter (Yang et al., 2012). Furthermore, LPS can delay gastric emptying by inducing COX-2 expression (Calatayud et al., 2002), leading to increased gastric pressure. The relaxation of the lower esophageal sphincter and increased gastric pressure can both aggravate gastroesophageal reflux, and long-term gastroesophageal reflux increases the risk of BE and esophageal cancer. Additionally, certain toxins produced by bacteria can cause DNA damage and promote the occurrence of tumors. For instance, several gram-negative bacteria, including *Escherichia coli*, *Actinobacillus actinomycetemcomitans* and Campylobacter, can produce cytotoxic swelling toxin (CDT), which can cause DNA damage, promoting the occurrence of cancer (Nesic et al., 2004; He et al., 2019). *F. nucleatum* can produce virulence factor Fad A, which promotes tumor development by interacting with E-cadherin to activate β-catenin signal (Rubinstein et al., 2013). Despite the increase of gram-negative bacteria, the diversity of the esophageal microflora in esophageal cancer tends to decline. Studies have found that different tumor types, and the presence or absence of lymph node metastasis can lead to significant changes in esophageal microflora, although there are different opinions on whether there are significant differences in esophageal microflora of different tumor stages. However, most studies believe that the esophageal microflora of esophageal tumors at different clinical stages varies. The load of gram-positive bacteria can be utilized as a prognostic indicator for esophageal cancer. *F. nucleatum* was significantly higher in tumor tissues compared to paired normal tissues, with a higher positive rate observed in tumors with metastasis compared to tumors without metastasis. This finding is consistent with the aforementioned virulence factor Fad A of *F. nucleatum*, which promotes tumor development by activating β-catenin signaling. While several mechanisms linking the esophageal microflora to esophageal diseases have been proposed (**Figure 2**), more research is needed to fully understand how these microorganisms interact with each other and with the host’s immune system in order to cause or exacerbate esophageal diseases. Moreover, there is ongoing debate about whether changes in the microbiota lead to the development of esophageal diseases, or if esophageal diseases drive the transformation of the microbiota. Esophageal diseases such as esophagitis, GERD, or esophageal cancer can cause changes in the esophageal mucosa and local environment, resulting in alterations in the esophageal microbiota. Studies have shown a dose-dependent association between penicillin exposure and an increased risk of cancer, including esophageal cancer (Boursi et al., 2015), providing support for the idea that changes in the microflora precede inflammation and disease. Other studies have shown that the imbalance of microflora is a potential side effect of acidic environment caused by GERD (Amir et al., 2014), *P.gingivalis* can selectively infect the ESCC subjects’ tumor and adjacent mucosa, but cannot infect the healthy mucosa of the control group (Gao et al., 2016), indicating that esophageal disease can lead to the changes of esophageal microflora. These researches suggest a reciprocal causal relationship between esophageal disease and changes in esophageal microflora, whereby the mucosal microenvironment can be remodeled by pathogenic microflora, and changes in the mucosal microenvironment can further promote alterations in the microflora. However, despite the increasing number of studies on esophageal microflora, achieving complete uniformity of results is challenging due to differences in inclusion and exclusion criteria, sampling methods, sampling sites, culture and determination methods, among others. Various limitations exist in current methods of studying esophageal microflora, including the invasiveness of mucosal biopsy, inability to successfully sample certain bacteria attached to the mucosa with esophageal aspirate, bias in traditional bacterial culture, and inability of gene amplification technology to assess bacterial viability or potential amplification bias after treatment steps in 16S rRNA gene sequencing. With the development of genomics, we can make use of the prospect of multi-omics, that is, meta-proteinomics, metatranscriptomics to know the expression of each gene and metabolomics to see the potential impact of metabolites of flora on the disease. In the near future, utilizing these advanced techniques may enable us to better understand the role of esophageal microbiome in esophageal diseases, identify effective molecular markers for predicting conditions such as BE, esophageal cancer, and other esophageal diseases, and develop more accurate screening and diagnostic plans to contribute to the prevention and treatment of esophageal diseases.

## Author contributions

QZ: data curation, formal analysis, writing-original draft. LF, XC and YQ: review, revision and editing. LX: resources, supervision, project administration and funding acquisition. All authors contributed to the article and approved the submitted version.
